# Ebola virus RNA detection on fomites in close proximity to confirmed Ebola patients; N’Zerekore, Guinea, 2015

**DOI:** 10.1371/journal.pone.0177350

**Published:** 2017-05-11

**Authors:** Romain Palich, Leonid M. Irenge, Eric Barte de Sainte Fare, Augustin Augier, Denis Malvy, Jean-Luc Gala

**Affiliations:** 1 Alliance for International Medical Action, Dakar, Senegal; 2 Center for Applied Molecular Technologies, Louvain Catholic University, Brussels, Belgium; 3 Department of Tropical Medicine and Clinical International Health, Division of Infectious and Tropical Diseases, University Hospital of Bordeaux, Bordeaux, France; 4 INSERM1219, University of Bordeaux, Bordeaux, France; Deutsches Primatenzentrum GmbH - Leibniz-Institut fur Primatenforschung, GERMANY

## Abstract

**Objective:**

Health care workers (HCWs) in contact with patients with Ebola virus disease (EVD) are exposed to a risk of viral contamination. Fomites contaminated with the patient’s blood or body fluids represents this risk. Our study aims to detect Ebola virus (EBOV) RNA within the high- and low-risk areas of an Ebola treatment unit (ETU) located in inland Guinea during the 2014–2015 West African Ebola epidemics. For samples from patients’ immediate vicinity, we aim to seek an association between viral RNA detectability and level of plasma viral load of patients (intermediate to high, or very high).

**Methods:**

Swabbing was performed on immediate vicinity of Ebola patients, on surfaces of an ETU, and on personal protective equipment (PPE) of HCWs after patient care and prior to doffing. All samples were assessed by quantitative reverse-transcribed PCR (RT-qPCR).

**Results:**

32% (22/68) of swabs from high-risk areas were tested positive for EBOV RNA, including 42% (18/43) from patients’ immediate vicinity, and 16% (4/25) from HCWs PPE. None of specimens from low-risk areas were tested positive (0/19). Swabs were much more often viral RNA positive in the vicinity of patients with a very high plasma viral load (OR 6.7, 95% CI [1.7–23.4]).

**Conclusion:**

Our findings show the persistence of EBOV RNA in the environment of Ebola patients and of HCWs, in a Guinean ETU, despite strict infection prevention and control measures. This detection raises the possibility that patients’ environment could be a potential source of contamination with the virus.

## Introduction

The recent Ebola virus disease (EVD) outbreak affecting West Africa was caused by a Zaire Ebola virus (EBOV) strain [1]. More of 28,000 EVD cases have been reported along with more of 11,000 deaths, from early 2014 to December 2015, in Guinea, Liberia and Sierra Leone, with unprecedented disastrous outcomes for countries and people, including the death of many health care workers (HCWs).

It has been shown that transmission of EBOV occurs through direct contact with ill or dead Ebola patients, or with their body fluids [2–5]. In this context, Ebola treatment units (ETUs) were set up to isolate patients from the community and to provide them with optimal care, and under infection prevention and control as a constant concern. Physical barriers were established within the ETUs in order to separate high-risk (where confirmed patients are cared for) from low-risk areas. Herein, strict procedures for cleaning with repetitive disinfection of surfaces as well as for wearing personal protective equipment (PPE) aimed to prevent the accidental contamination of HCWs [6,7]. Within the high-risk area, many measures were implemented to limit both cross-contamination between patients and spread of the virus by HCWs: respect of a suitable distance between patients, screens between beds, organized turn-over and circuit for HCWs during care, hands disinfection before caring a new patient, limited number of patients per ward.

So far, few data are available on surface contamination by EBOV. During the Ebola epidemic that occurred in 2000 in Uganda, Bausch et al. sampled different areas of an ETU as well as PPE [8]. All were negative for EBOV RNA detection by reverse transcription-PCR (RT-PCR), except the specimen from contaminated gloves and intravenous insertion sites. Much more recently in Sierra Leone, Poliquin et al. sampled the different areas of an ETU setting and of PPE used too [9]. This study showed the persistence of EBOV RNA from material in contact with patients, and on gloves of HCWs, before decontamination.

The aim of our study was to assess the presence of EBOV RNA in the near vicinity of EVD patients in an ETU, on PPE from HCWs after care, and in other high- and low-risk areas, and to seek an association between the persistence of EBOV RNA in the patient’s vicinity and the level of their concurrent EBOV plasma viral load.

## Methods

The study was conducted from January 19th to February 11th, 2015, in the setting of N’Zerekore ETU (inland Guinea), managed by the French non-governmental organization (NGO) Alliance for International Medical Action (ALIMA) (S1 Fig and S1 File).

### Swabs collection

Swabs were collected from high- and low-risk areas. Comprehensive details on the different ETU areas are given in S1 File, including an N’Zerekore ETU map. In high-risk area, swabs were collected from immediate vicinity of patients (IV insertion site, patient’s skin, mattress, clothes, blanket, digestive losses bucket, IV drip stand, floor), used PPE from HCWs. In this respect, PPE picture with swab location is provided in S1 File too.

Each swab was opened and moistened with 3 drops of sterile 0.9% sodium chloride solution in the area of interest. Environmental samples were collected in the close vicinity of Ebola-infected patients (from mattresses, clothing and blankets, buckets for stool or vomit, IV pools and the floor) at least six hours after the previous cleaning of the ward. Swabs were scrubbed methodically against the surface to be sampled, placed into tubes and sealed. In addition, concurrent blood samples were collected from each patient for determination of EBOV plasma viral load, the same day of environmental sampling. Sampling on PPE was carried out directly after nursing and medical care and before disinfection of hands. Human samples consisted of non-invasive oral and skin swab samples (intravenous line (IV) insertion sites, mouth, armpits, and from a leg wound). Negative control swabs (N = 12) consisted of swabs opened in the high- or the low-risk area, moistened with sterile 0.9% NaCl solution, and directly placed into collection tubes.

### Samples analysis

Swabs and plasma samples were processed in the N’Zerekore ETU associated field laboratory, and assessed by real time RT-PCR for RNA of EBOV detection, using the RealStar Filovirus Screen RT-PCR kit 1.0 (Altona Diagnostics, Hamburg, Germany), according to the manufacturer’s instructions. The threshold cycle (Ct) was used as a measure of EBOV RNA concentration in the specimen. EBOV RNA detection from swab was positive for Ct<40 (limit of detection). Viral load in plasma samples was categorized as very high (Ct <20) or intermediate to high (Ct ≥20) (see Technical Appendix for details on samples collection and analysis).

### Ethical considerations

The N'zerekore ETU participated in the JIKI study (evaluation of the efficacy of favipiravir during EVD), approved by several Ethics Committee, as described elsewhere [10]. All patients signed a written consent for this study, including blood sampling. Besites, blood samples referred to the study investigation were samples carried out for routine care. No additional blood samples were carried out. Swabs collection was specifically approved by the Institutional Review Board of Université catholique de Louvain/Saint-Luc Hospital (Belgium). For swabs, oral consent were obtained in the native language of each patient. All results were anonymized.

## Results

**Fig 1:**
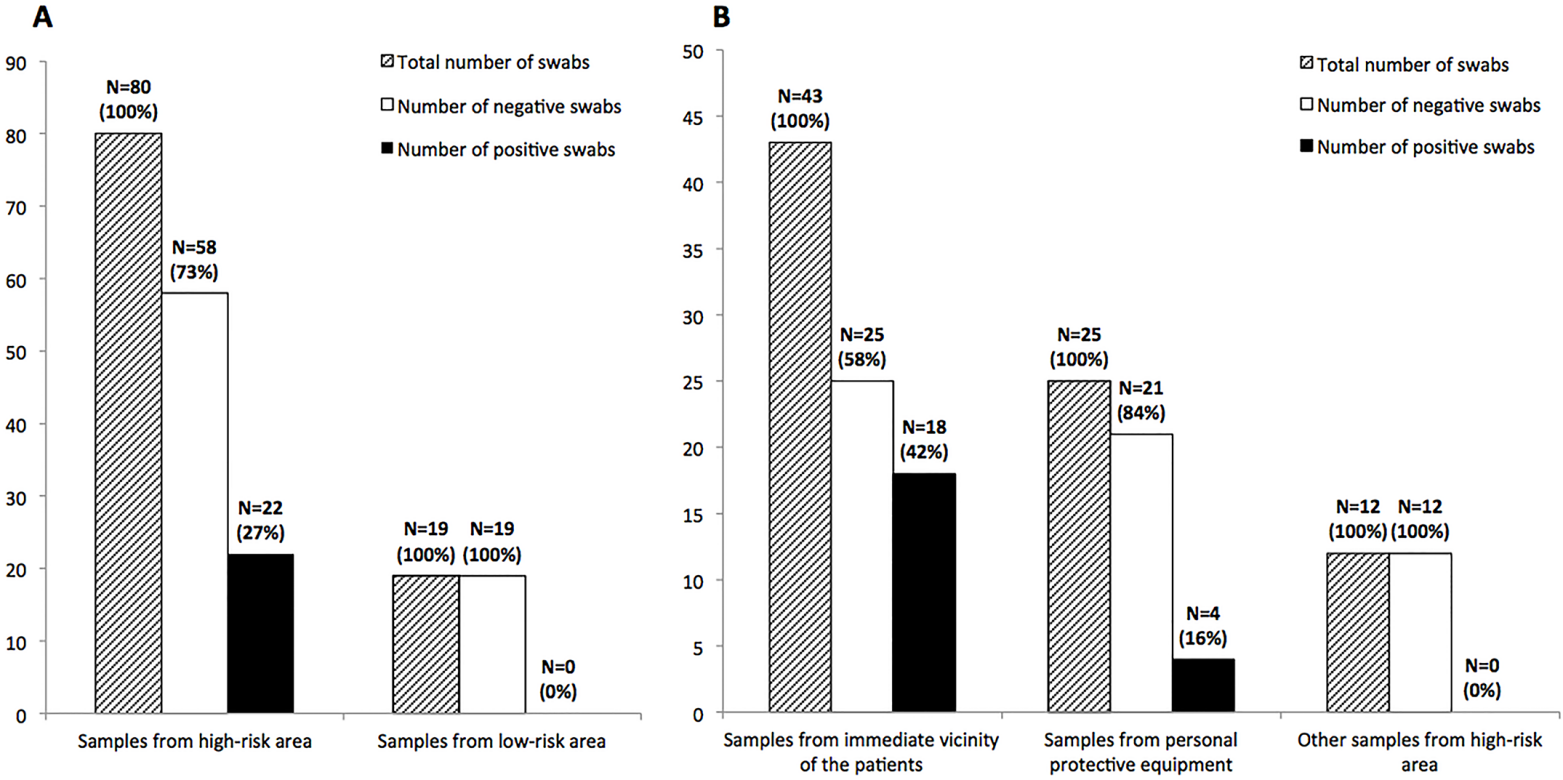
Number of swabs from low- and high-risk areas. A. Number of positive (EBOV RNA+) and negative (EBOV RNA-) swabs from high-risk area. B. Number of positive (EBOV RNA+) and negative (EBOV RNA-) swabs from low-risk area.

A total of 99 swabs were collected; 80 from high-risk areas (43 from the immediate vicinity of confirmed patients, 25 from PPE of HCWs, and 12 from other high-risk areas) and 19 from low-risk areas (Fig 1).

In the immediate vicinity of patients, 41.9% (18/43) of the specimens were tested positive (Fig 1A and Table 1). These included 50% (9/18) of the swabs taken on the patients’ body surface. Three samples (two from an IV insertion site and the third from the wound) appeared to have absorbed fresh blood or to contain dried blood and all were positive for EBOV RNA. For swabs collected from fomites in direct contact with an infected patient (mattress, clothes, blanket, bucket for digestive losses), 41.2% (7/17) were positive. One swab collected on a mattress appeared to be contaminated with fresh blood, two swabs from clothes and blankets contained dried blood and another mattress swab was clearly contaminated by stool. All these contaminated swabs were positive for EBOV RNA. The six specimens collected outside and inside the stool buckets were all negative, in spite of traces of stool on two of the swabs. Regarding the other swabs collected in the immediate vicinity of patients, only two were positive; both looked bloody and were taken from the floor in the immediate vicinity of a patient.

**Table 1:** Samples from the immediate vicinity of the patients.

Sample	Number of swabs	Color	RT-PCR result (Ct ± SD)	Patient ID	Viral load (Plasma Ct)
IV insertion site	7				
1/7		Clear	Negative	A	23.3
2/7		Clear	Negative	B	20.4
3/7		Clear	Negative	C	16.3
4/7		Clear	Negative	D	20.5
5/7		Clear	Positive (33.96 ± 0.48)	E	16.6
6/7		Pink†	Positive (26.97 ± 0.07)	F	16.6
7/7		Red‡	Positive (21.80 ± 0.20)	G	18.7
Mouth (inner surface of lower lip)	4				
1/4		Clear	Negative	A	23.3
2/4		Clear	Positive (29.98 ± 0.10)	D	20.5
3/4		Clear	Positive (29.81 ± 0.08)	B	20.4
4/4		Clear	Positive (26.40 ± 0.00)	E	16.6
Armpit skin	3				
1/3		Clear	Negative	A	23.3
2/3		Clear	Positive (33.23 ± 0.25)	F	16.6
3/3		Clear	Positive (27.99 ± 0.07)	D	20.5
Abdominal skin	3				
1/3		Clear	Negative	A	23.3
2/3		Clear	Negative	B	20.4
3/3		Clear	Negative	D	20.5
Wound (leg)	1	Pink‡	Positive (29.91 ± 0.12)	E	16.6
Mattress	7				
1/7		Clear	Negative ^b^	C	16.3
2/7		Clear	Negative ^b^	H	28.4
3/7		Clear	Positive (40.35 ± 4.04) ^b^	F	16.6
4/7		Brown	Positive (32.31 ± 0.70) ^b^	D	20.5
5/7		Clear	Positive (27.88 ± 0.17) ^b^	A	23.3
6/7		Brown	Positive (23.69 ± 0.11) ^b^	E	16.6
7/7		Red‡	Positive (22.48 ± 0.15) ^b^	G	18.7
Clothes / blanket	4				
1/4		Clear	Negative	A	23.3
2/4		Clear	Negative	E	16.6
3/4		Pink†	Positive (25.81 ± 0.06)	D	20.5
4/4		Pink†	Positive (25.04 ± 0.04)	G	18.7
Bucket digestive losses (inside)	2				
1/2		Brown	Negative^c^	B	20.4
2/2		Brown	Negative^c^	D	20.5
Bucket digestive losses (outside)	4				
1/4		Clear	Negative	A	23.3
2/4		Clear	Negative	G	18.7
3/4		Clear	Negative	F	16.6
4/4		Grey	Negative	D	20.5
IV drip stand	3				
1/3		Clear	Negative	B	20.4
2/3		Clear	Negative	C	16.3
3/3		Clear	Negative	H	28.4
Floor (patient’s immediate vicinity)	5				
1/5		Grey	Negative ^b^	A	23.3
2/5		Grey	Negative ^b^	A	23.3
3/5		Grey	Negative ^b^	H	28.4
4/5		Brown	Positive (23.70 ± 0.13)^d^	G	18.7
5/5		Red	Positive (20.94 ± 0.05)^a^	G	18.7
**TOTAL, number of positives (%)**	**43**		**18/43 (41.9)**		

^†^ Dried blood.

^‡^ Fresh blood.

^a.^ Before spraying with the 0.5% hypochlorite solution.

^b.^ After spraying with the 0.5% hypochlorite solution.

^c.^ Buckets contain 1–2 liters of 0.5% hypochlorite solution.

^d.^ Ten minutes after spraying with the 0.5% hypochlorite solution.

**Fig 2:**
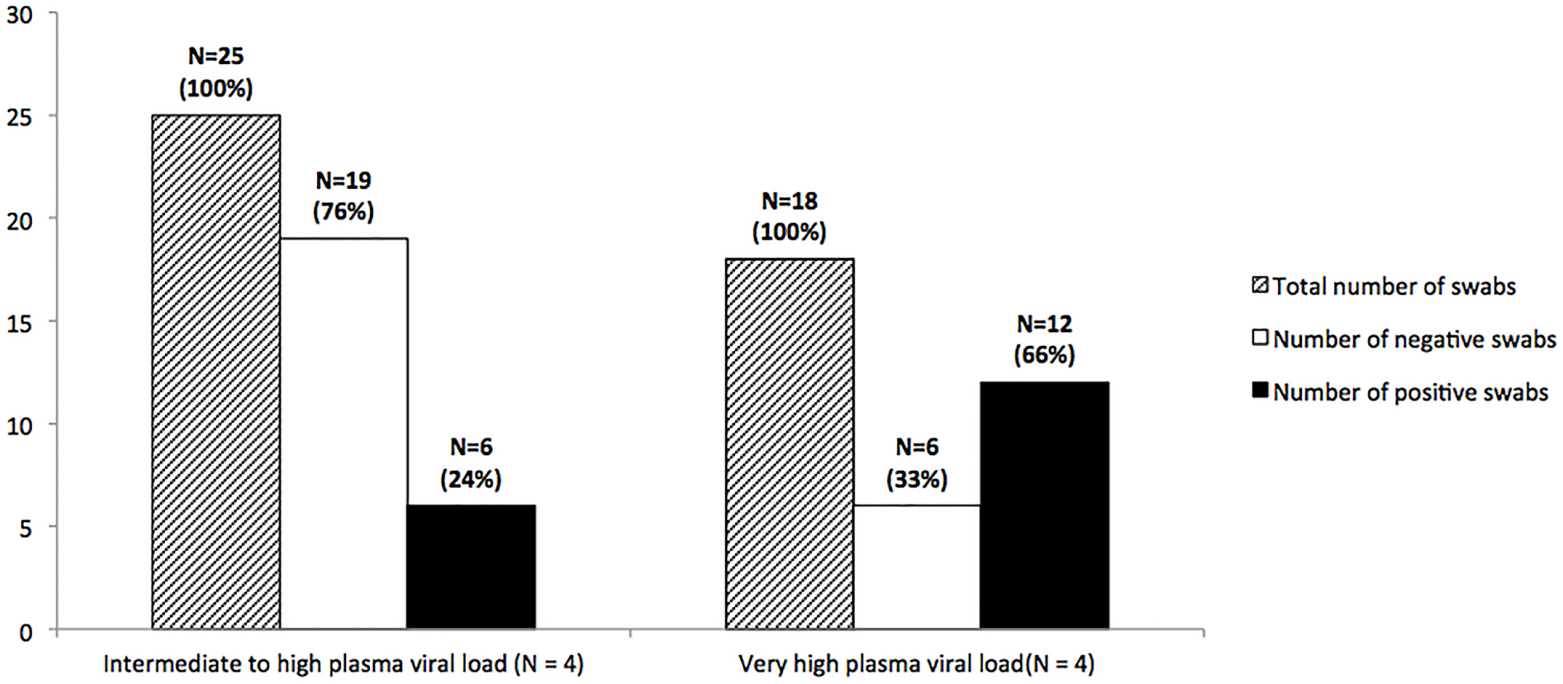
Number of positive (EBOV RNA+) and negative (EBOV RNA-) swabs from close proximity of patients with intermediate to high or very high EBOV plasma viral load.

Eight patients provided plasma samples and plasma viral load, as estimated by Ct, ranged from 16.3 Ct to 28.4 Ct (Table 1). For the 43 swabs collected in the immediate vicinity of one among the eight patients, that particular patient presented a very high viral load (Ct<20) for eighteen swabs (41.9%) and an intermediate to high viral load (Ct≥20) for the remaining 25 swabs (58.1%) (Fig 2). No positive swab was obtained from the patient harbouring the highest plasma viral load (Patient C: Ct = 16.3) or from the patient with the lowest plasma viral load (Patient H: Ct = 28.4). At least one positive swab was obtained from the six remaining patients. Altogether, 67% (12/18) of these positive swabs were collected on or close to patients with a very high plasma viral load (Ct<20; OR 6.7, 95%CI[1.7–23.4]), whereas only 24% (6/25) of positive swabs came from patients with an intermediate to high plasma viral load (Ct≥20; OR 0.16, 95%CI[0.04–0.61]).

Of the 25 swabs collected from PPE, four (16%) were found positive for EBOV RNA (Fig 1B and Table 2). Two of these specimens showed evidence of contamination by fresh blood.

**Table 2:** Samples from personal protective equipment in high-risk area.

Sample	Number of swabs	Color	RT-PCR result (Ct ± SD)
Physicians’ gloves	4		
1/4		Clear	Negative
2/4		Clear	Negative
3/4		Clear	Negative
4/4		Pink	Positive (29.16 ± 0.07)
Nurses’ gloves	6		
1/6		Clear	Negative
2/6		Clear	Negative
3/6		Clear	Negative
4/6		Clear	Negative
5/6		Clear	Positive (30.58 ± 0.04)
6/6		Pink	Positive (27.54 ± 0.12)
Apron	3		
1/3		Clear	Negative
2/3		Clear	Negative
3/3		Clear	Negative
Protective suit	4		
1/4		Clear	Negative
2/4		Clear	Negative
3/4		Clear	Negative
4/4		Clear	Positive (33.27 ± 0.35)
Hood	2		
1/2		Grey	Negative
2/2		Grey	Negative
Goggles	3		
1/3		Clear	Negative
2/3		Clear	Negative
3/3		Clear	Negative
Breathing mask	3		
1/3		Clear	Negative
2/3		Clear	Negative
3/3		Clear	Negative
**TOTAL, number of positives (%)**	**25**		**4/25(16)**

Other swabs collected within the high-risk area and within the low-risk area were negative (Fig 1B and S1 and S2 Tables), as well as negative control swabs.

## Discussion

Using real-time RT-PCR-based analysis of swabs collected in the N’Zerekore ETU, Guinea, we show that EBOV RNA persistence could be detected from the immediate vicinity of patients and from used PPE. Indeed, 32.7% (18/55) swabs from high-risk area and 16% (4/25) swabs from PPE were positive; all swabs from low-risk area were negative.

These results are consistent with data published in 2007 and 2016, by our colleagues in Uganda and in Sierra Leone [8,9].

All swabs visibly soiled with body fluids were positive, with the exception of two swabs from stool buckets; this finding is consistent with the reported effectiveness of EBOV inactivation by sodium hypochlorite [6]. Some specimens visibly clean were conversely tested positive, probably linked with recontamination after a previous cleaning and disinfection of the surface.

Although EBOV RNA positive swabs were found in the environment of most infected patients, swabs appeared much more often positive in the environment of patients with a very high plasma viral load (and low Ct). Patients with highest plasma viral load are those in the most advanced wet-stage of EVD [10], and probably those with the largest production of highly contaminating execrates. This observation certainly explains our findings, and could be of great importance for the triage of patients, suggesting secretory patients should be ascertained and even separated from others pending the result of diagnostic test.

The main limit of our study is the lack of viral culture as a confirmation of the viability and infectivity of EBOV from RNA positive specimens. Working in the field in Guinea does not allow to perform viral culture. In addition, in the middle of the epidemic we did not have the opportunity to send abroad biological samples, in part for safety reasons and mainly for legal reasons. This point has been discussed by Poliquin et al., who also did not process viral culture [9], and by Bausch et al., who questioned the reliability of a negative viral culture from two RNA EBOV positive samples [8].

A previous study showed that EBOV RNA persists beyond the presence of the infectious agent itself [11]. Therefore we cannot conclude that the detected EBOV RNA from our samples matches the infectious virus. However: 1) failing to detect RNA EBOV probably reflects the absence of virus, and 2) detecting RNA EBOV should allow to reinforce preventive measures in identified areas. This statement is supported by Youkee, et al., who showed that sodium hypochlorite disinfection drastically reduces the detection of EBOV RNA in the patients’ environment [12]. Besides, several contaminations occurred in HCWs despite preventions measures. The conclusion is that there are still numerous gaps in knowledge about contamination from Ebola patients’ environment.

Spengler, et al. showed that viral culture could be negative from blood of patients with low plasma viral loads (Ct>30) [13]. Thus, EBOV RNA could better reflect the presence of virus in case of low inoculum.

Single strand viral RNA material is relatively stable in the environment, especially in high temperatures and moisty conditions as it is the case in Guinea and un other tropical settings. A recent study demonstrated persistent viability of EBOV on surfaces and in fluids for several days under simulated environmental conditions of an ETU for the climate of West Africa [14].

All these elements argue for the detection of EBOV RNA as a surrogate marker of the presence of Ebola virus in the ETU environment in West Africa.

To conclude, our study provides useful data for the management of patients and HCWs, in the EVD context. Further investigations with systematic sample collection should be however conducted to correlate the detection of EBOV genetic material to the presence and infectivity of the virus.

## Supporting information

S1 Fig N’Zerekore ETU map. The high-risk area is red, and the low-risk area is orange and green. The high-risk area includes the triage area (A), the EVD-suspected wards (B), the EVD-confirmed wards (C), the mortuary (D). The dressing area (E) allowed to enter in the high-risk area, and the undressing area (F) allowed to come back in the low-risk area. The low-risk area includes the locker room (G), the doctor’s and hygienists’ offices (H), the pharmacy (I), the laundry (J), the showers and WCs (K), the social workers’ office (L) and the lunch area (M). Number of swabs collected from the different areas: A (2), B (1), C (50), D (2), E (1), F (27), G (3), H (5), I (1), J (1), K (3), L (1) and M (2).
(TIF)

S2 Fig Personal protective equipment (PPE) picture. Number of swabs collected from the different parts of PPE: hood (2), goggles (3), breathing mask (3), protective suit (4), gloves (10) and apron (3).
(TIF)

S1 File N’Zerekore ETU organization. (PDF)

S2 File Virological investigation and analysis of specimens. (PDF)

S1 Table Other samples from the vicinity of the patients. (PDF)

S2 Table Samples from low-risk area. (PDF)
