# Supplementary figures and images for: Ebola virus RNA detection on fomites in close proximity to confirmed Ebola patients; N’Zerekore, Guinea, 2015
Source: PLoS One. 2017 May 11;12(5):e0177350. doi: 10.1371/journal.pone.0177350 (PMC5426669; doi:10.1371/journal.pone.0177350)

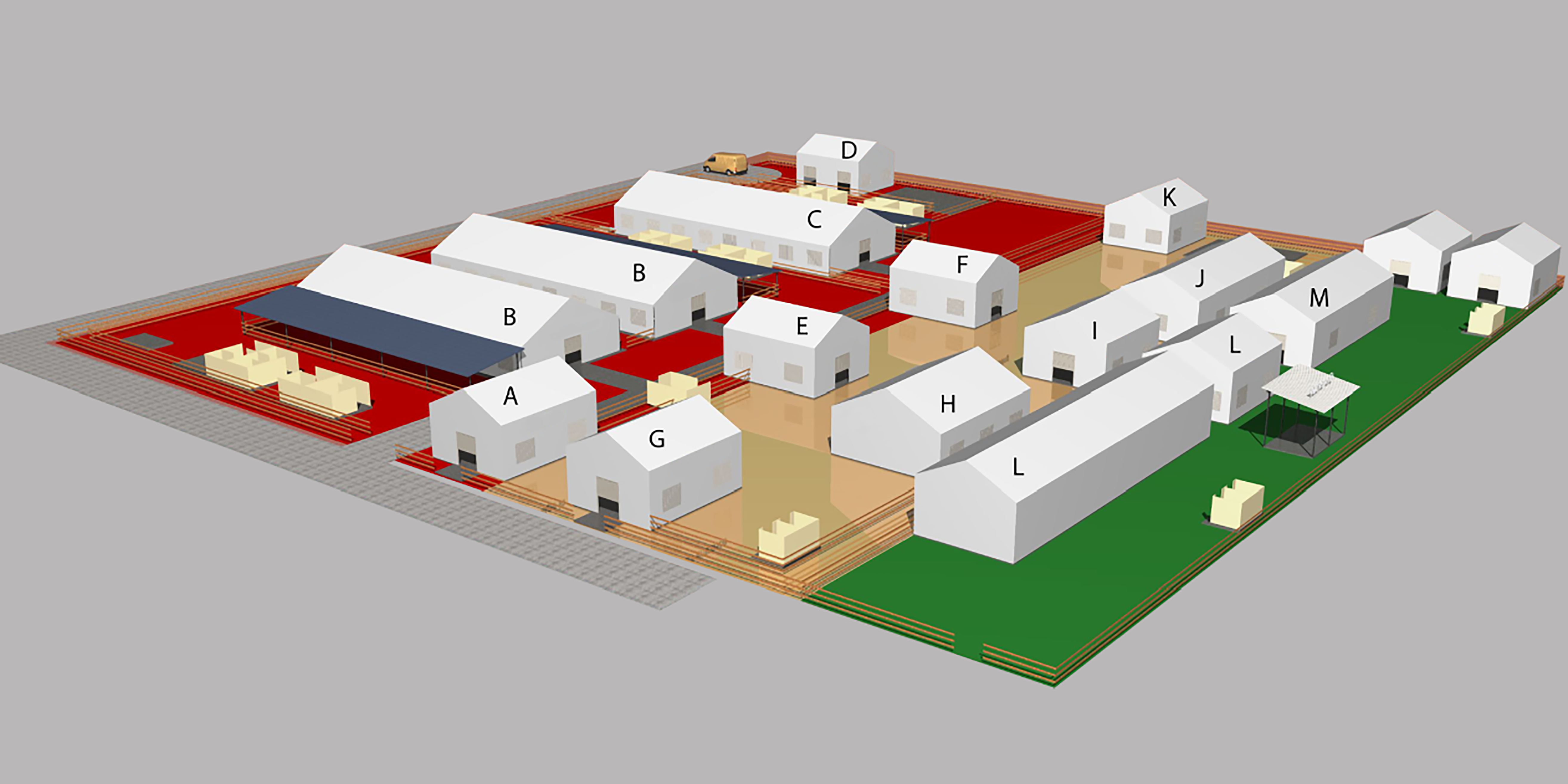

Supplement: S1 Fig — The high-risk area is red, and the low-risk area is orange and green. The high-risk area includes the triage area (A), the EVD-suspected wards (B), the EVD-confirmed wards (C), the mortuary (D). The dressing area (E) allowed to enter in the high-risk area, and the undressing area (F) allowed to come back in the low-risk area. The low-risk area includes the locker room (G), the doctor’s and hygienists’ offices (H), the pharmacy (I), the laundry (J), the showers and WCs (K), the social workers’ office (L) and the lunch area (M). Number of swabs collected from the different areas: A (2), B (1), C (50), D (2), E (1), F (27), G (3), H (5), I (1), J (1), K (3), L (1) and M (2). (TIF) [file pone.0177350.s001.tif]

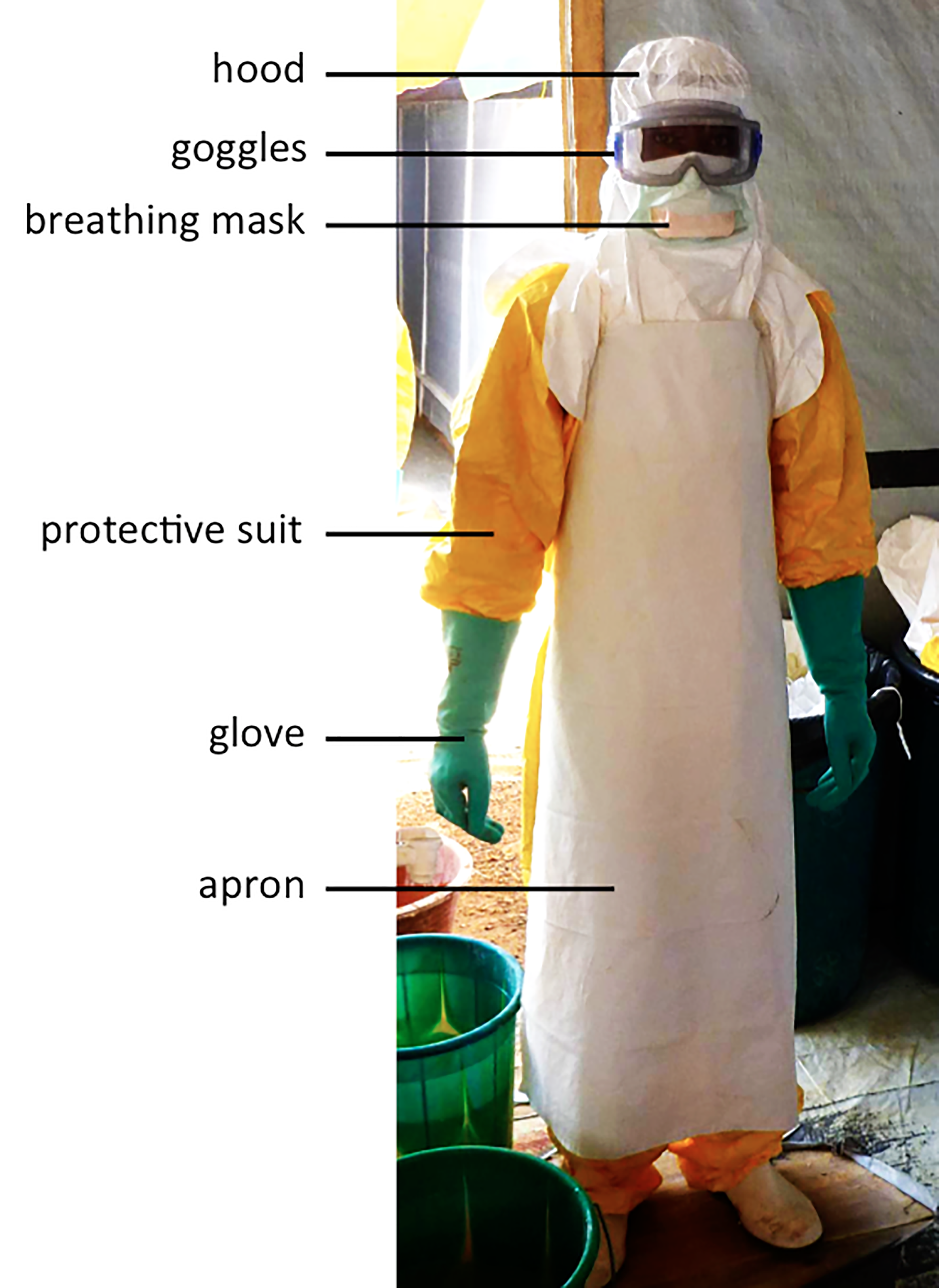

Supplement: S2 Fig — Number of swabs collected from the different parts of PPE: hood (2), goggles (3), breathing mask (3), protective suit (4), gloves (10) and apron (3). (TIF) [file pone.0177350.s002.tif]
