# Supplementary material for: Ebola virus RNA detection on fomites in close proximity to confirmed Ebola patients; N’Zerekore, Guinea, 2015
Source: PLoS One. 2017 May 11;12(5):e0177350. doi: 10.1371/journal.pone.0177350 (PMC5426669; doi:10.1371/journal.pone.0177350)
Supplement: S1 File — (PDF) [file pone.0177350.s003.pdf]

S1 File. N’Zerekore ETU organization.

The district of N’Zerekore is located in the forest south-east of the country. The N’Zerekore ETU was built according to established guidelines implemented in nearly all ETUs in Guinea, Liberia and Sierra Leone. The N’Zerekore Ebola treatment unit (ETU) is divided into a low- and high-risk zone. The low-risk zone consists of medical and nursing administrative tents, a laundry area, storage tents, water chlorination points and other facilities to support the high-risk zone which, in turn, consists of a series of tents dedicated to triage and admission of suspect, probable or confirmed cases tents, a morgue and a high-risk waste area. A plan of the N’Zerekore ETU (with the location of the sampled swabs) is provided in S1 Fig. The PPE donning procedure is performed in the low-risk zone. Entering the high-risk zone implies wearing PPE permanently. The doffing is performed at the edge of the high-risk zone. The HCW only leaves the high-risk zone after several disinfection steps and the used PPE is either destroyed without leaving the high-risk zone (for example, combination suits and gloves) or plunged in an 0.5% hypochlorite solution before evacuation through a dedicated ‘dirty waste’ circuit for recycling (for example, boots, masks and aprons). Patients are initially assessed in the triage area and, after first clinical assessment, are relocated to the tents for suspect or probable cases while awaiting results from the biological test.
The ETU opened on 2nd December, 2014, one year after the EBOV outbreak began. Overall, 145 patients were admitted to the ETU during the epidemic, of whom 76 were tested positive for Ebola virus. In these confirmed cases with EBOV infection, the mortality rate was up to 60.8%. Around 190 people worked daily within the N’Zerekore ETU, including 12 doctors, 22 nurses, 17 nurse assistants, 10 social workers, and 130 cleaners. Entries of HCWs into the high-risk area were frequent, with as many as 100 entry/exit procedures per day in the tracking log book. Guidelines for the use of PPE were strictly followed. The patient ward was disinfected daily with a detergent and a 0.5% hypochlorite solution. Beds and mattresses were disinfected with 0.5% hypochlorite solution before each admission. In addition, the ETU guideline required the immediate spraying of body fluids with 0.5% hypochlorite solution. All clothing and bedding were burned when soiled or after the patient was discharged from the ward. Running tap water at a concentration of 0.5% hypochlorite was available inside the ward for hands (in this case, glove washing) and reusable medical devices (thermometers, blood pressure meters and cuffs).
