# Supplementary material for: Ebola virus RNA detection on fomites in close proximity to confirmed Ebola patients; N’Zerekore, Guinea, 2015
Source: PLoS One. 2017 May 11;12(5):e0177350. doi: 10.1371/journal.pone.0177350 (PMC5426669; doi:10.1371/journal.pone.0177350)
Supplement: S2 File — (PDF) [file pone.0177350.s004.pdf]

S2 File. Virological investigation and analysis of specimens.

Blood and swab samples were processed in a portable negative pressure glove box (OWR GmbH, Elztal, Germany) and assessed by reverse-transcription real-time PCR (RT-qPCR) for detection of EBOV. Plasma samples were obtained for determination of viremia. Blood was collected in EDTA tubes and immediately decanted for 30 minutes (for plasma and blood cells separation). Plasma was subsequently used for analysis. An inactivation step, followed by RNA extraction was performed using Qiagen reagents (Hilden, Germany) according to the manufacturer's protocol. In brief, 50 µL of RNAse and DNAse-free PCR-grade water were added to 90 µL of plasma sample. The 140 µL mixture was added to a tube containing 560 µL of AVL buffer in the glovebox. The solution was incubated at room temperature inside the glovebox during 10 minutes for viral inactivation. This inactivation step was completed by adding 560 µL of ethanol 100%. The mixture was subsequently removed from the glovebox and total RNA extraction and purification was performed outside the glovebox, according to the manufacturer's protocol. Final elution was carried out in 60 µL of AVE buffer (Qiagen, Hilden, Germany). Swab samples were stirred in 140 µL of RNAse and DNAse-free water and the total RNA extraction process and reverse-transcription was the same as described above for plasma samples.

EBOV RNA was assayed using semi-quantitative EBOV RT-qPCR (RealStar© Zaire ebolavirus RT-PCR Kit 1.0, Altona Diagnostics, Hamburg, Germany) according to the manufacturer's instructions. Each extracted swab or plasma sample was assayed in triplicate per RT-qPCR assay. The RT-qPCR assay was carried out on the CFX96 real-time platform (Bio-Rad, Nazareth, Belgium) using 31 µL of a reaction mixture which contained 10 µL of extracted RNA as template, and 21 µL of a mixture composed of 5 µL of Master A, 15 µL of Master B and 1 µl of internal control from the same manufacturer. It is of note that Master A and B reagents contain all components (buffer, enzymes, primers, and probes) to allow reverse transcription, PCR mediated amplification and target detection in one reaction setup. Cycling times and temperatures were used as recommended by Altona Diagnostics.

The RT-qPCR results were expressed in terms of cycle threshold (Ct), which is inversely proportional to the EBOV RNA content of the sample. The sample was considered to be positive for EBOV RNA if an exponential fluorescence signal was generated in each well, with a Ct cut-off value <40, and if both internal control and positive controls generated the expected exponential fluorescent signals with an intra-assay coefficient of variation between the three wells of the same sample $<5\%$. A Ct of 20 is corresponding to $\sim 10^8$ genome copies/ml.
