# Supplementary material for: Ebola virus RNA detection on fomites in close proximity to confirmed Ebola patients; N’Zerekore, Guinea, 2015
Source: PLoS One. 2017 May 11;12(5):e0177350. doi: 10.1371/journal.pone.0177350 (PMC5426669; doi:10.1371/journal.pone.0177350)
Supplement: S1 Table — (PDF) [file pone.0177350.s005.pdf]

S1 Table. Other samples from the vicinity of the patients.

Sample	Number of swabs	Color	RT-PCR result
Table (confirmed ward)	1	Grey	Negative
Medical waste bin (confirmed ward)	2		
1/2		Clear	Negative
2/2		Clear	Negative
Clear water tap (confirmed ward)	1	Clear	Negative
Patients' shower (confirmed ward)	1	Grey	Negative
Patients' WC 1 (confirmed ward)	1	Clear	Negative
Patients' WC 2 (confirmed ward)	1	Grey	Negative
Mortuary	2		
Floor		Grey	Negative
Death cover		Clear	Negative[a]
Suspected ward (floor)	1	Grey	Negative
Triage area (floor)	1	Grey	Negative
Undressing area (floor)	1	Grey	Negative
**TOTAL, number of positives (%)**	**12**		**0/12 (0)**

NOTES. a. After spraying with the 0.5% hypochlorite solution.
