# Supplementary material for: Ebola virus RNA detection on fomites in close proximity to confirmed Ebola patients; N’Zerekore, Guinea, 2015
Source: PLoS One. 2017 May 11;12(5):e0177350. doi: 10.1371/journal.pone.0177350 (PMC5426669; doi:10.1371/journal.pone.0177350)
Supplement: S2 Table — (PDF) [file pone.0177350.s006.pdf]

S2 Table. Samples from low-risk area.

Sample	Number of swabs	Color	RT-PCR result
Doctors' office			
Desks	1	Grey	Negative
Computer keyboards	1	Clear	Negative
Paper records	1	Clear	Negative
Alert phone	1	Clear	Negative
Hygienists' office (desks)	1	Grey	Negative
Social workers' office (desks)	1	Grey	Negative
Locker room	2		
Lockers for dirty clothes		Grey	Negative
Lockers for dirty boots		Grey	Negative
Common thermometer	1	Clear	Negative
Dressing area (floor)	1	Grey	Negative
Undressing area (floor)	1	Grey	Negative
Triage area (floor)	1	Grey	Negative
Pharmacy (desk)	1	Clear	Negative
Laundry (floor)	1	Grey	Negative
Shower	1	Clear	Negative
WC 1	1	Clear	Negative
WC 2	1	Clear	Negative
Lunch area	2		
Table		Clear	Negative
Clear water tap		Clear	Negative
**TOTAL, number of positives (%)**	**19**		**0/19(0)**
